# Attentional Tuning Resets after Failures of Perceptual Awareness

**DOI:** 10.1371/journal.pone.0060623

**Published:** 2013-04-02

**Authors:** Paul E. Dux, Warrick Roseboom, Christian N. L. Olivers

**Affiliations:** 1 School of Psychology, The University of Queensland, St Lucia, Queensland, Australia; 2 Human Information Science Laboratory, Nippon Telegraph and Telephone Corporation Communication Science Laboratories, Atsugi, Kanagawa, Japan; 3 Department of Cognitive Psychology, VU University, Amsterdam, The Netherlands; Cuban Neuroscience Center, Cuba

## Abstract

Key to successfully negotiating our environment is our ability to adapt to current settings based on recent experiences and behaviour. Response conflict paradigms (e.g., the Stroop task) have provided evidence for increases in executive control after errors, leading to slowed responses that are more likely to be correct, and less susceptible to response congruency effects. Here we investigate whether failures of perceptual awareness, rather than failures at decisional or response stages of information processing, lead to similar adjustments in visual attention. We employed an attentional blink task in which subjects often fail to consciously register the second of two targets embedded in a rapid serial visual presentation stream of distractors, and examined how target errors influence performance on subsequent trials. Performance was inferior after Target 2 errors and these inter-trial effects were independent of the temporal lag between the targets and were not due to more global changes in attention across runs of trials. These results shed light on the nature of attentional calibration in response to failures of perceptual consciousness.

## Introduction

To overcome our severe information processing capacity limitations and interact successfully with the visual world, we must select relevant over irrelevant information from an environment that constantly changes across time and space. This task is performed primarily by the attentional system and it is for this reason that the mechanisms underlying attention are a topic of intense interest to psychologists and neuroscientists [1].

A key question is whether and how attention learns from its mistakes and successes. There is an extensive literature suggesting that attention tightens its hold over cognitive processing after an error has been made, or even after the potential for an error arises (so called “high conflict” trials) [2], [3], [4], [5], [6], [7], [8]. Typical paradigms used in these investigations include the Stroop [9], Simon [10], and Eriksen Flanker [11] tasks, in which the target stimulus is accompanied by a competing stimulus (or feature) that evokes either the same (congruent) or a different (incongruent) response, with reaction time (RT) the preferred measure. After an error, the next response is typically slowed, more likely to be correct, and response congruency effects on RTs are reduced. As such, the cognitive control literature has almost exclusively looked at errors in response selection and its subsequent effects on resolving response conflict. Moreover, the nature of the post-error adaptation (suppressing the wrong response or slowing down responses in general) is likely to reflect a substantial amount of executive control, rather than changes in selective attention.

Here we are interested in how attention adapts to perceptual errors rather than response errors. What happens if an observer is asked to look for a target which he or she knows is there, but then fails to become aware of? Will the current attentional settings be further strengthened, similar to the tightening of cognitive control at the response end? Or will new settings be adopted – because the previous settings failed to provide the target – in the hope that these will result in better performance next time? To answer these questions, we employ rapid serial visual presentation (RSVP), which has been used widely to study attention. In RSVP, stimuli appear one after the other in the same spatial location for a fraction of a second each [12]. A particularly important RSVP phenomenon is the attentional blink (AB) [13], which refers to subjects' impaired ability to report the second of two target stimuli (T1 and T2) in an RSVP stream if they appear within 200–500 ms of one another. Thought to reflect a failure of perceptual awareness, the AB has been hypothesised to be the result of either attentional resource depletion [14], [15], [16], [17], [18] or attentional selection mechanisms [19], [20], [21]. Recently, Dux and Marois [22] have argued that both of these factors play an important role in generating the AB [23], [24], [25].

The RSVP task, and especially the dual-target version wherein the AB is revealed, provides a useful tool for investigating how attention adapts to failures of perceptual awareness. This is because the type of error made is typically a complete failure to consciously register the stimulus, rather than to select the wrong response [26]. Identification or detection accuracy is therefore the preferred measure, rather than response speed. A second reason is that the AB task usually generates many such errors (a quarter to half the number of trials is not uncommon), thus providing us with the necessary power for assessing post-perceptual-error adaptations in attention. To our knowledge, no study has looked at how an error on one trial affects performance on the next in the AB task. A recent article by Yashar and Lamy [27] reported faster RTs to a target in an RSVP stream when its features (and those of the distractors) matched those from the previous trial, relative to when they were switched. This suggests that when a target is successfully selected, an attentional set for its features is maintained or strengthened, at the expense of the other target. However, this study says relatively little about how failures of conscious perception affects later performance, as each trial only contained one, above-threshold target. Moreover, as RTs were the primary measure, post-perceptual processes may have contributed.

Although there is no direct evidence for inter-trial effects on perceptual awareness in RSVP, there is empirical work showing that pre-trial cognitive operations influence performance on the AB. For example, Olivers and Nieuwenhuis [28] found that when individuals viewed a positive image (e.g., a puppy) prior to performing a dual-target RSVP task the AB was reduced relative to when viewing neutral or negative images [29]. In addition, when subjects were instructed to adopt a more “diffuse” attentional state prior to undertaking an AB task, the second target deficit was reduced relative to conditions where subjects were told to give the RSVP streams maximal attention [30], [31]. Thus, the pre-trial configuration of the attentional network appears to play a central role in consciousness and the AB. Convergent evidence for this comes from recent work using electroencephalogram (EEG) which has shown that pre-trial brain activity is correlated with AB performance. Specifically, MacLean and Arnell [32] found the alpha band activity (10–12 Hz) prior to the onset of a dual-target RSVP stream was suppressed and this reduction was more pronounced in trials where T2 was missed as opposed to reported accurately [33]. Crucially, this effect was only observed if T2 appeared within the AB temporal window, with the opposite effect observed when the second target was presented outside this window. Similarly, Pincham and Szücs [34] found that T2 performance in an AB task was predicted by a positive deflection in the event-related potential (ERP) that occurred approximately 200 ms before the trial. Collectively, these findings support the idea that attentional settings pre-trial, as reflected by the ongoing-state of the brain before stimulus presentation, play a significant role in determining which items enter consciousness.

Here we investigate the influence of inter-trial dynamics on perceptual awareness using a standard dual-target RSVP (AB) paradigm. Specifically, we assess how performance on T2 in a previous trial determines T2 performance on a subsequent trial, and what this tells us about how attention adapts. To preview the results, target detection/identification is facilitated following T2 correct trials and this effect is independent of potential global changes in task performance (due to practice or random fluctuations in attention) and the previous trial's lag.

## Experiment 1

Experiment 1 employed a standard dual-target RSVP protocol where subjects searched for two digit targets, that appeared amongst letter distractors, separated by a temporal lag of 200 (lag 2) or 800 ms (lag 8). Of key interest was the effect of previous trial (n-1) T2 accuracy on current trial (n) performance. We also examined the effect of the T1-T2 lag on trial n-1, as this provided a manipulation of the attentional demands on the previous trial (with the short lag trials being more difficult than the long lag trials), regardless of whether an error was made. This allowed us to dissociate error-based adaptations from difficulty per se.

### Materials & Method

#### Ethics Statement

All subjects gave informed written consent and The University of Queensland Ethics Review Committee approved the experimental protocol.

#### Subjects

Thirty-six undergraduate students from The University of Queensland participated for course credit. Two were excluded, one for having T2 accuracy at floor and the other for being at ceiling on this measure. The final sample consisted of 26 females with a mean age of 21.41 years (standard deviation  = 3.5 years).

#### Stimuli, Apparatus & Procedure

Subjects each completed three runs of a categorical dual-target RSVP search task, where the targets were digits drawn from the set 2–9 and the distractors letters from the alphabet excluding I, L, O, Q, U, V. Twenty-two items appeared in each RSVP stream. Trials began with a fixation square in the centre of the screen for 500 ms, following this, the RSVP stream appeared, with each item being presented for 100 ms and subtending approximately 1° of visual angle. The fixation square, RSVP items and report prompts were presented in black and the background was white. No stimulus was repeated in the stream and the text was presented in Courier New font. T1 was presented at serial position 3, 5, 7 or 9 (randomly assigned for each trial) and T2 appeared at lag 2 (200 ms stimulus onset asynchrony between T1 and T2) or lag 8 (800 ms). Each run contained 200 trials and the 2 lags were presented equally often across the run. Subjects pressed the space bar to begin runs and subsequent trials began immediately after the target identities were entered after a prompt at the end of the RSVP stream (e.g., “Enter First Target Identity”). There was no time pressure on the response. The experiment was programmed in MATLAB® (The MathWorks, Natick, MA) using the psychophysics toolbox extension [35], [36] and was presented using a Sony Trinitron 21-inch flat screen cathode ray tube (CRT) monitor with a Apple Macintosh mini computer running OS ×10.5.

### Results & Discussion

Each trial (trial n) was sorted based on the lag and T2 accuracy (conditional on T1 being accurate) on the preceding trial (trial n-1). As is standard in the AB literature, only trials where T1 was correct were included in the analysis (i.e., T2|T1 correct), however the pattern of results was identical when the data (for both trial n and trial n-1) were not conditional on T1 performance. Trials were not sorted based on preceding T1 accuracy given that the high performance on this dependent variable limited the number of n-1 incorrect trials.

**Figure 1:**
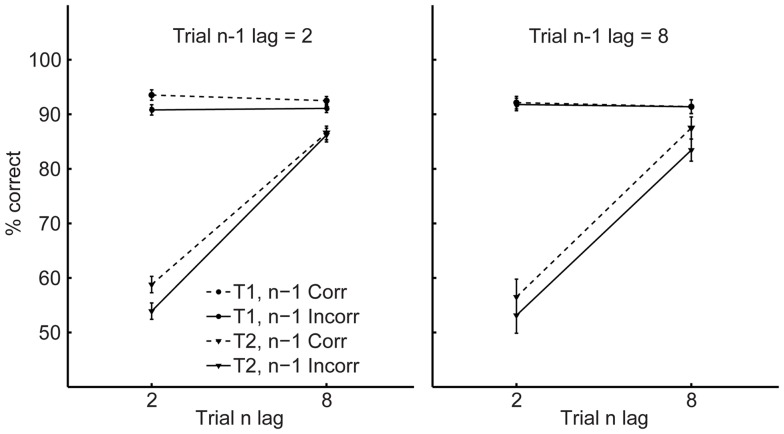
Experiment 1 results. T1 and T2 accuracy as a function of current lag (trial n, 2 vs. 8), previous lag (trial n-1, 2 vs. 8), and previous trial T2 accuracy (trial n-1, correct vs. incorrect). Error bars represent standard error of the difference between trials preceded by correct and incorrect responses.

Figure 1 shows T1 and T2 performance on the current trial (n) as a function of current lag (trial n, 2 vs. 8), previous lag (trial n-1, 2 vs. 8), and previous T2 accuracy (trial n-1, correct vs. incorrect). A 2×2×2 repeated measures ANOVA with these factors demonstrated that for both T1 and T2, independent of the lag on trial n and n-1, performance was superior following correct relative to incorrect trials (main effects of trial n-1 T2 accuracy  = *Fs*>4.5, *ps*<0.05). The only other significant result was the main effect of trial n lag on T2 accuracy, *F*(1, 33) = 82.6, *p*<0.001, indicating an overall AB. Thus, the present results demonstrate that after a correct response subjects are more likely to be accurate on the next trial, but the previous trial's lag does not influence performance on the subsequent trial.

These findings suggest that failure to consciously perceive a given event can have a significant effect on the perception of subsequent events, even if the latter events appear to be independent. Specifically, here we find that an error in an AB task is more likely to lead to another error. This effect does not appear to reflect the demand for cognitive control on the previous trial, as has typically been found in response time paradigms such as the Stroop task [4]. In fact, while performance typically improves in the Stroop task after difficult (i.e., incongruent and incorrect) trials, here performance did not depend on previous lag, even though lag itself had a clear and substantial effect on performance.

The results provide a first indication that observers adapt their attentional settings on a trial-by-trial basis in the RSVP paradigm, and that these changes are triggered by a failure of conscious perception. However, note that the results may also stem from random fluctuations or slow-wave changes in attention throughout a run of trials (i.e., based on both external and internal factors that are not related to the task). As long as these changes are less rapid than the rate at which trials are presented, such fluctuations also predict accuracy to correlate across consecutive trials, simply because consecutive trials occur closely in time. Experiment 2 was designed to rule out this alternative account.

## Experiment 2

In Experiment 1 we found that first and second target RSVP performance was enhanced following a trial where a correct response was made on T2 relative to an incorrect response. However, the crucial data was correlational in nature, as the selection of T2 correct and incorrect trials was made post-hoc, and not manipulated, as is typically the case for an experimental factor. Indeed, the results did not demonstrate that T2 accuracy on a given trial *caused* a correct or incorrect response on the subsequent one. Thus, the data might be due to a third factor, such as fluctuations in attentional state, but also practice effects. To wit, as one goes through an experiment performance improves and, therefore, correct responses are more likely to follow correct responses.

Here we tested this alternate account by making subjects believe that they made a mistake on some trials. On each trial, we provided feedback on target report. Crucially, and unbeknownst to the subjects, we inserted catch trials where only T1 was presented and subjects were informed that their T2 response was incorrect. In this respect, the substantial number of target incorrect trials in the dual-target RSVP paradigm is an advantage, as it made the catch trials unnoticeable. The catch trials were randomly interspersed throughout the experiment. If our inter-trial effects reflect changes other than those caused by the previous trial (e.g., practice, or random attentional fluctuations) then performance following these trials should be better than performance following a true error (as they appear randomly throughout the runs). Alternatively, if our findings reflect adaptations in attentional settings, conditional on previous trial behaviour, then performance after catch trials should be the same as that after true error trials.

### Materials & Method

The method for the second experiment was identical to that for Experiment 1 except where specified.

#### Subjects

Twenty-six new subjects participated in this experiment (17 females, mean age  = 21.62 years, standard deviation  = 5.84 years).

#### Stimuli, Apparatus & Procedure

Fifty percent of trials were lag 2 trials, 40% lag 8 trials and 10% were catch trials where T2 was replaced with a distractor. Subjects were not informed regarding the existence of these catch trials; rather they were instructed that two targets were always present in each stream. Catch trials were distributed randomly in the runs of trials and appeared on average 9 trials apart (average minimum number of trials between catch trials  = 0, sequential presentation, average maximum  = 43). The RSVP streams contained 21 items (one or two targets and 20 or 19 distractors). At the end of each trial feedback was given for each target with either “Correct” being presented in green or “Incorrect” in red. On catch trials, feedback of “Incorrect” was always given for the T2 response. The other stimuli were presented in white on a grey background. These changes were made to the colours of the stimuli, relative to Experiment 1, in order to equate the salience/contrast of the green and red feedback signals. Subjects were presented with 600 trials and could take breaks at the 25%, 50% and 75% points in the experiment. Afterwards, subjects were questioned regarding whether they had noticed that there was no T2 at all on some trials.

### Results & Discussion

**Figure 2:**
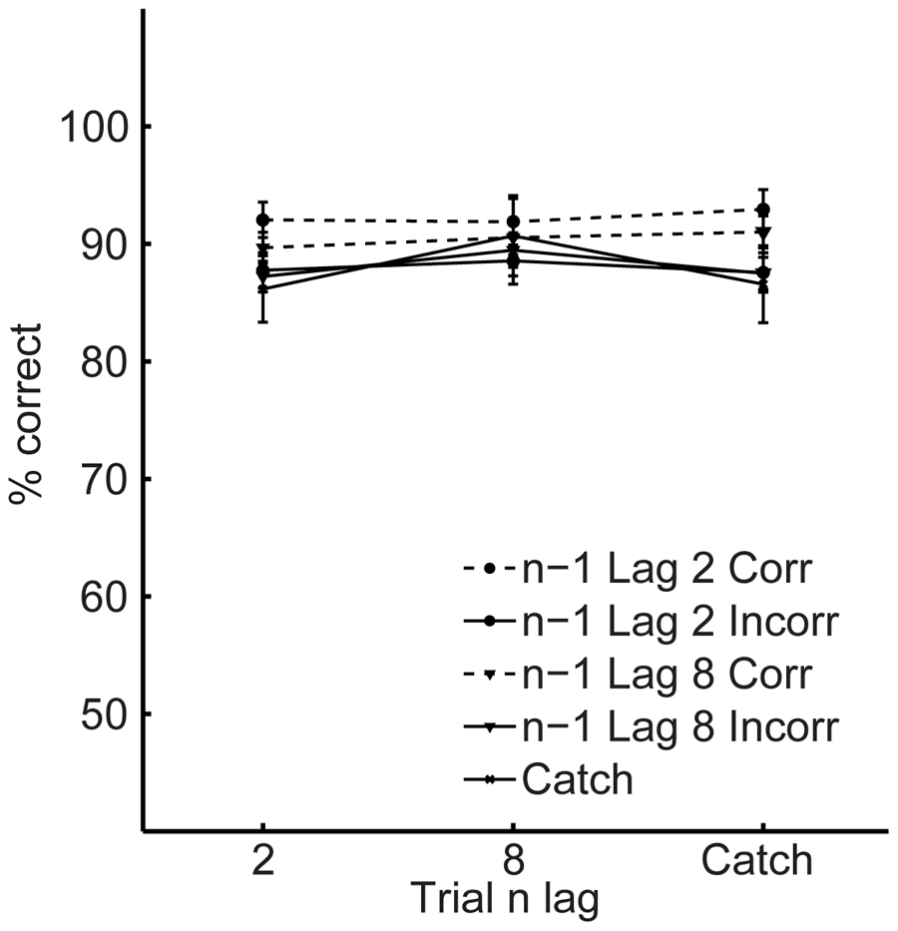
First target accuracy in Experiment 2. T1 accuracy as a function of current lag (trial n, 2 vs. 8 vs. Catch), previous lag (trial n-1, 2 vs. 8 vs. Catch), and previous T2 accuracy (trial n-1, correct vs. incorrect). On catch trials no second target was presented, but an incorrect response was displayed. Error bars represent standard error of the difference between trials preceded by correct and incorrect responses.

Figures 2 (T1) and 3 (T2) show the key results and the dependent variables were identical to those used in Experiment 1 (again, conditionalising T2 accuracy on T1 report did not influence the pattern of results). An examination of the data, sorted on the basis of n-1 T2 accuracy (given T1 correct) and n-1 lag revealed findings, for both T1 and T2 performance, that were identical to those from Experiment 1. Two identical 2 (trial n lag: 2 vs. 8)×2 (trial n-1 T2 accuracy: correct vs. incorrect) ×2 (trial n-1 lag: 2 vs. 8) repeated measures ANOVAs demonstrated that performance on T1 and T2 was superior after correct T2 report in the preceding trial relative to incorrect report (main effects of trial n-1 T2 accuracy, *Fs*>5.7, *p*<0.05). The only other main effect was that of trial n lag on T2 accuracy, *F*(1, 25) = 51.1, *p*<0.001, indicating an overall AB. Importantly, for both T1 and T2 analyses, identical main effects of trial n-1 T2 accuracy were found when the lag 2 incorrect trials were replaced with catch trials in the ANOVAs (*Fs*>6.03, *ps*<0.05). In addition, T1 and T2 accuracy following catch trials was no different to that after incorrect trials at lag 2 (*ts*<1.5, *ps*>0.15). Thus, the inter-trial effects from Experiment 1, and replicated again here, are the direct consequence of what happened on the previous trial, and do not reflect a third factor such as a practice effect or slow-wave fluctuations in attentional state.

**Figure 3:**
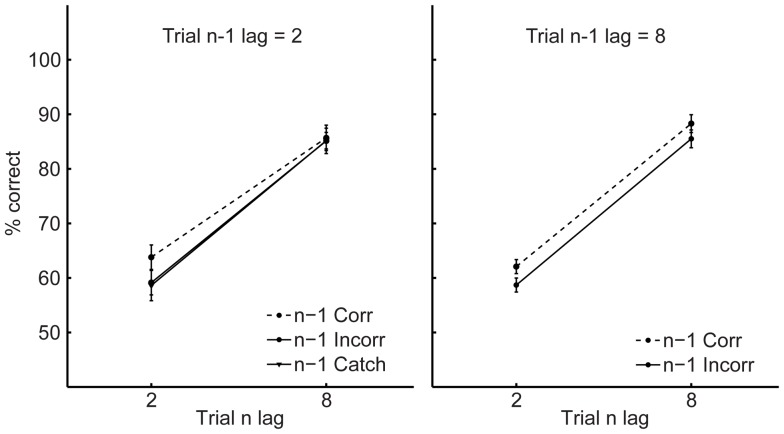
Second target accuracy in Experiment 2. T2 accuracy as a function of current lag (trial n, 2 vs. 8), previous lag (trial n-1, 2 vs. 8 vs. Catch), and previous T2 accuracy (trial n-1, correct vs. incorrect). Error bars represent standard error of the difference between trials preceded by correct and incorrect responses.

## General Discussion

The present study investigated the influence of previous failures of perceptual consciousness on attentional processing in subsequent trials. We presented subjects with dual-target RSVP steams, where failures of perceptual consciousness are likely to occur, and examined how performance on trial n-1 affected that on trial n. Previous work had focused on such inter-trial effects in RT decision making and response conflict tasks [4], [5], [8], however to our knowledge no study had assessed how previous trial perceptual performance influenced behaviour on subsequent trials.

In Experiment 1, we observed that trial n performance was superior following correct T2 report on trial n-1, relative to when errors were made on the previous trial. This demonstrates that there are indeed inter-trial effects in paradigms tapping conscious perception. Experiment 2 showed that this result was not due to a practice effect or other global changes in processing. This was the case as performance following catch trials (those without a second target, where subjects were given feedback that they had made an error) was impaired to the same extent as that following true error trials. In addition, our inter-trial effects were not contingent on feedback being given to subjects as an identical pattern of results was found between Experiment 1 and 2 even though only the latter experiment included feedback.

Based on the findings of these two experiments we hypothesise that subjects try to fine-tune their attentional settings on a trial-by-trial basis by sticking to their current set when it is successful, but shifting away from it when an error occurrs, in search for a better setting. Thus, accuracy is superior after a correct response because similar successful attentional settings are employed on the following trial, but impaired after an error because new settings are adopted which are likely non-optimal for the upcoming task. A similar mechanism may underlie enhanced selective attention following monetary rewards [37]. Recently it has been shown that accurately identifying a target in RSVP can alter visual perception as measured using the tilt-after effect [38]. Pascucci and Turatto argued that this is because successfully detecting a target provides an endogenously generated reward. Thus, in the present experiments subjects may maintain their attentional settings after a correct response because it is rewarding.

The results have implications for theories of temporal attention and consciousness as they demonstrate that pre-trial behaviour, which likely affects attentional/cognitive control settings, influences dual-target RSVP processing. Several recent models of the AB, such as the Overinvestment/Boost and Bounce theory of Olivers and colleagues [20], [39] and the Threaded Cognitive Framework [40], hypothesise that the phenomenon does not reflect information processing capacity limits [22], but rather imprecise cognitive control settings. The present results certainly suggest that pre-trial attentional settings influence the dual-target RSVP processing, however we found that none of the experiments provided evidence that the AB itself was influenced by previous trial performance (just overall T2 performance) as lag did not interact with the presence of errors on the previous trial. Thus, more research is required before a definitive statement can be made regarding the influence of inter-trial behaviour on the AB itself. These implications aside, we emphasise though that the goal of the present work was not testing theories of the AB. Rather we used the AB as a tool to a) probe attention after failures of perceptual awareness (rather than response errors), and b) to induce sufficient numbers of errors in the first place (something that is not always the case in standard response time tasks).

### Error monitoring and adjustments of control

As discussed above, our pattern of results was different to that typically observed in decision making/response interference paradigms using RT as the main measure. In these studies performance is usually less error-prone after difficult trials, or after errors and this, together with increased RTs, has been taken as evidence that control is ramped up in response to demanding conditions [2], [4], [5], [8]. In the present study, performance was not affected by difficulty (as induced by lag on trial n-1), nor was it improved after errors, rather it was impaired. These opposing results can be reconciled if we take into account the differences between our task and those that have been used so far in the error monitoring literature.

For one, the error monitoring literature has almost exclusively looked at response conflict and response error, as induced by Stroop, Eriksen, Flanker, Simon, and Stopping tasks, as well as paradigms with response deadlines [2], [3], [4], [6], [7], [8]. As far as we are aware, we are the first to look at the effects of *perception errors* on subsequent performance. It is possible that different mechanisms underlie post-perceptual-error adjustment and post-response-error adjustment. Consistent with this hypothesis, Van Veen, Cohen, Botvinick, Stenger and Carter [41] and Milham et al. [42], only found activation of the anterior cingulate cortex (ACC; a medial frontal lobe brain region thought to be involved in conflict monitoring and providing a signal for cognitive control) for target stimuli that were accompanied by a conflicting response competitor, not by mere perceptual competitors. Others have found activity for perceptual competitors, but in a different area, namely the dorsolateral prefrontal cortex [42], [43]. Finally, Verbruggen, Notebaert, Liefooghe and Vandierendonck [44] used an Eriksen flanker task and found adjustments in conflict resolution (as measured through a reduced flanker congruency effect on the subsequent trial) after perceptual conflict, but this was a rather small effect when compared to the adjustments occurring after response conflict. As were found in the same experiment.

It should also be noted, that while response errors may indeed be followed by tightened control, this tends to go at the expense of response *speed* - known as post-error slowing [8]. That is, subjects become more cautious after an error, which indeed leads to a smaller chance of making another error. But without a clear and quantifiable speed/accuracy trade-off function this does not warrant the conclusion that performance as a whole improves after an error. Of course, this type of control is not possible at the perceptual end, as perception cannot be strategically postponed (not unless one closes one's eyes).

We propose that perceptual inter-trial effects, such as the one described here, are dominated by learning mechanisms, where successful target selection leads to a reinforcement of the representations that led to that selection [45], thus increasing the chances of successful selection in the future. Errors, on the other hand, lead to a weakening or abandonment of the current attentional set, which becomes disadvantageous if the attentional settings were already quite optimal. The idea that attention tries to rapidly learn from experience is consistent with the literature on inter-trial effects in standard visual search. Under these spatial search conditions, *priming of pop-out* is also observed, with successful selection of a target feature (e.g., a red target) leading to more efficient selection of that same target on the next trial, even if observers know that the target is more likely to be of a different type (e.g. green) [46], [47]. These effects occur automatically, and can operate over a considerable time frame with inter-trial effects being observed between trials separated by 8–10 others.

## Conclusions

We have demonstrated that previous trial perception influences subjects' ability to consciously register a target in a subsequent trial. This inter-trial effect appears to reflect the dynamics associated with the trial-to-trial updating of attentional settings, and as such it is the first demonstration of learning on the basis of perceptual misses, rather than response errors. Future work should aim to further characterise the mechanisms that underlie this adjustment of settings to facilitate awareness and examine the extent to which they tap the same processes previously implicated in other inter-trial phenomena.

## References

[1] Itti L, Rees G, Tsotsos JK,editors (2005) Neurobiology of Attention. Amsterdam:Elsevier/Academic Press.

[2] Botvinick M, Nystrom LE, Fissell K, Carter CS, Cohen JD (1999) Conflict monitoring versus selection-for-action in anterior cingulate cortex. Nature 402: 179–181. 10647008 10.1038/46035

[3] Gratton G, Coles MGH, Donchin E (1992) Optimizing the use of information: Strategic control of activation and responses. Journal of Experimental Psychology: General 121: 480–506. 1431740 10.1037//0096-3445.121.4.480

[4] Kerns KG, Cohen JD, MacDonald AW, Cho RY, Stenger VA, et al (2004) Anterior cingulate conflict monitoring and adjustments in control. Science 303: 1023–1026. 14963333 10.1126/science.1089910

[5] MacDonald AW, Cohen JD, Stenger VA, Carter CS (2000) Dissociating the role of the dorsolateral prefrontal and anterior cingulate cortex in cognitive control. Science 288: 1835–1838. 10846167 10.1126/science.288.5472.1835

[6] Stürmer B, Leuthold H, Soetens E, Schröter H, Sommer W (2002) Control over location-based priming in the Simon task: Behavioral and electrophysiological evidence. Journal of Experimental Psychology: Human Perception and Performance 28: 1345–1363. 12542132 10.1037//0096-1523.28.6.1345

[7] Verbruggen F, Logan GD (2009) Proactive adjustments of response strategies in the stop-signal paradigm. Journal of Experimental Psychology: Human Perception and Performance 35: 835–854. 19485695 10.1037/a0012726 PMC2690716

[8] Ridderinkhof KR, Ullsperger M, Crone EA, Nieuwenhuis S (2004) The role of the medial frontal cortex in cognitive control. Science 306: 443–447. 15486290 10.1126/science.1100301

[9] Stroop JR (1935) Studies of interference in serial verbal reactions. Journal of Experimental Psychology 28: 643–662.

[10] Simon JR, Rudell AP (1967) Auditory S-R compatibility: the effect of an irrelevant cue on information processing. Journal of Applied Psychology 51: 300–304. 6045637 10.1037/h0020586

[11] Eriksen BA, Eriksen CW (1974) Effects of noise letters upon the identification of a target letter in a nonsearch task. Perception & Psychophysics 16: 143–149.

[12] Potter MC, Levy EI (1969) Recognition memory for a rapid sequence of pictures. Journal of Experimntal Psychology 81: 10–15. 10.1037/h0027470 5812164

[13] Raymond JE, Shapiro KL, Arnell KM (1992) Temporary suppression of visual processing in an RSVP task: An attentional blink? Journal of Experimental Psychology: Human Perception & Performance 18: 849–860. 1500880 10.1037//0096-1523.18.3.849

[14] Chun MM, Potter MC (1995) A two-stage model for multiple target detection in rapid serial visual presentation. Journal of Experimental Psychology: Human Perception and Performance 21: 109–127. 7707027 10.1037//0096-1523.21.1.109

[15] Jolicœur P, Dell'Acqua R (1998) The demonstration of short-term consolidation. Cognitive Psychology 36: 138–202. 9721199 10.1006/cogp.1998.0684

[16] Dell'Acqua R, Jolicœur P, Luria R, Pluchino P (2009) Reevaluating encoding-capacity limitations as a cause of the attentional blink. Journal of Experimental Psychology: Human Perception and Performance 21: 109–127. 10.1037/a0013555 19331492

[17] Dux PE, Asplund CL, Marois R (2009) Both exogenous and endogenous target salience manipulations support resource depletion accounts of the attentional blink: A reply to Olivers, Spalek, Kawahara & Di Lollo. Psychonomic Bulletin & Review 16: 219–224. 19543438 10.3758/PBR.16.1.219 PMC2699278

[18] Dux PE, Asplund CL, Marois R (2008) An attentional blink for sequentially presented targets: Evidence in favor of resource depletion accounts. Psychonomic Bulletin & Review 15: 809–813. 18792508 10.3758/pbr.15.4.809 PMC3644218

[19] Di Lollo V, Kawahara J, Ghorashi SMS, Enns JT (2005) The attentional blink: Resource depletion or temporary loss of control. Psychological Research 69: 191–200. 15597184 10.1007/s00426-004-0173-x

[20] Olivers CNL, Meeter M (2008) A boost and bounce theory of temporal attention. Psychological Review 115: 836–863. 18954206 10.1037/a0013395

[21] Wyble B, Bowman H, Nieuwenstein M (2009) The attentional blink provides episodic distinctiveness: Sparing at a cost. Journal of Experimental Psychology: Human Perception and Performance 35: 324–337. 19485692 10.1037/a0013902 PMC2743522

[22] Dux PE, Marois R (2009) The attentional blink: A review of data and theory. Attention, Perception & Psychophysics 71: 1683–1700. 10.3758/APP.71.8.1683 PMC2915904 19933555

[23] Dell'Acqua R, Dux PE, Wyble B, Jolicœur P (2012) Sparing from the attentional blink is not spared from structural limitations. Psychonomic Bulletin & Review 19: 232–238. 22215469 10.3758/s13423-011-0209-3

[24] Kawahara J, Enns JT, Di Lollo V (2006) The attentional blink is not a unitary phenomenon. Psychological Research 70: 405–413. 16344995 10.1007/s00426-005-0007-5

[25] Martens S, Wyble B (2010) The attentional blink: Past, present and future of a blind spot in perceptual awarenes. Neuroscience & Biobehavioral Reviews 34: 947–957. 20025902 10.1016/j.neubiorev.2009.12.005 PMC2848898

[26] Sergent C, Dehaene S (2004) Is consciousness a gradual phenomenon? Evidence for an all-or-none bifurcation during the attentional blink. Psychological Science 15: 720–728. 15482443 10.1111/j.0956-7976.2004.00748.x

[27] Yashar A, Lamy D (2010) Intertrial repetition facilitates selection in time: Common mechanisms underlie spatial and temporal search. Psychological Science 23: 243–251. 10.1177/0956797609357928 20424053

[28] Olivers CNL, Nieuwenhuis S (2006) The beneficial effects of additional task load, positive affect, and instruction on the attentional blink. Journal of Experimental Psychology: Human Perception and Performance 32: 364–379. 16634676 10.1037/0096-1523.32.2.364

[29] Jefferies LN, Smilek D, Eich E, Enns JT (2008) Emotional valence and arousal interact in the control of attention. Psychological Science 19: 290–295. 18315803 10.1111/j.1467-9280.2008.02082.x

[30] Olivers CNL, Nieuwenhuis S (2005) The beneficial effect of concurrent task irrelevant mental activity on temporal attention. Psychological Science 16: 265–269. 15828972 10.1111/j.0956-7976.2005.01526.x

[31] Arend I, Johnston S, Shapiro K (2006) Task-irrelevant visual motion and flicker attenuate the attentional blink. Psychonomic Bulletin & Review 13: 600–607. 17201358 10.3758/bf03193969

[32] MacLean MH, Arnell KM (2011) Greater attentional blink magnitude is associated with higher levels of anticipatory attention as measured by alpha event-related desynchronization (ERD). Brain Research 1387: 99–107. 21362413 10.1016/j.brainres.2011.02.069

[33] Zauner A, Fellinger R, Gross J, Hanslmayr S, Shapiro K, et al (2012) Alpha entrainment is responsible for the attentional blink phenomenon. Neuroimage 63: 674–686. 22796984 10.1016/j.neuroimage.2012.06.075 PMC3459095

[34] Pincham H, Szűcs D (2012) Conscious access is linked to ongoing brain state: Electrophysiological evidence from the attentional blink. Cerebral Cortex 22: 2346–2353. 22079924 10.1093/cercor/bhr314

[35] Brainard DH (1997) The psychophysics toolbox. Spatial Vision 10: 433–436. 9176952

[36] Pelli DG (1997) The videotoolbox software for visual psychophysics: transforming numbers into movies. Spatial Vision 10: 437–442. 9176953

[37] Della Libera C, Chelazzi L (2006) Visual selective attention and the effects of monetary rewards. Psychological Science 17: 222–227. 16507062 10.1111/j.1467-9280.2006.01689.x

[38] Pascucci D, Turatto M (in press) Immediate effect of internal reward on visual adaptation. Psychological Science. 10.1177/0956797612469211 23670884

[39] Olivers CNL, van der Stigchel S, Hulleman J (2007) Spreading the sparing: Against a limited-capacity account of the attentional blink. Psychological Research 71: 126–139. 16341546 10.1007/s00426-005-0029-z

[40] Taatgen NA, Juvina I, Schipper M, Borst J, Martens S (2009) Too much control can hurt: A threaded cognition model of the attentional blink. Cognitive Psychology 59: 1–29. 19217086 10.1016/j.cogpsych.2008.12.002

[41] Van Veen V, Cohen JD, Botvinick MM, Stenger VA, Carter CS (2001) Anterior cingulate cortex, conflict monitoring, and levels of processing. NeuroImage 14: 1302–1308. 11707086 10.1006/nimg.2001.0923

[42] Milham MP, Banich MT, Webb A, Barad V, Cohen NJ, et al (2001) The relative involvement of anterior cingulate and prefrontal cortex in attentional control depends on nature of conflict. Cognitive Brain Research 12: 467–473. 11689307 10.1016/s0926-6410(01)00076-3

[43] Liu X, Banich MT, Jacobson BL, Tanabe JL (2006) Functional dissociation of attentional selection within PFC: response and non-response related aspects of attentional selection as ascertained by fMRI. Cerebral Cortex 16: 827–834. 16135781 10.1093/cercor/bhj026

[44] Verbruggen F, Notebaert W, Liefooghe B, Vandierendonck A (2006) Stimulus and response conflict-induced cognitive control in the flanker task. Psychonomic Bulletin & Review 13: 328–333. 16893003 10.3758/bf03193852

[45] Roelfsema PR, Van Ooyen A, Watanabe T (2010) Perceptual learning rules based on reinforcers adn attention. Trends in Cognitive Sciences 14: 64–71. 20060771 10.1016/j.tics.2009.11.005 PMC2835467

[46] Maljkovic V, Nakayama K (1994) Priming of pop-out: I. Role of features. Memory & Cognition 22: 657–672. 7808275 10.3758/bf03209251

[47] Olivers C, Humphreys G (2002) When visual marking meets the attentional blink: More evidence for top-down, limited-capacity inhibition. Journal of Experimental Psychology: Human Perception and Performance 28: 22–42.

